# Trimethoprim‐induced drug reaction with eosinophilia and systemic symptoms (DRESS) associated with reactivation of human herpes virus‐6 (HHV‐6) leading to acute liver failure

**DOI:** 10.1002/ccr3.3218

**Published:** 2020-08-09

**Authors:** Mohsan Subhani, Victor Dong, Aveen Connolly, Jonathan Salisbury, Rosa Miquel, Sarah Walsh, Tasneem Pirani

**Affiliations:** ^1^ Liver Intensive Therapy Unit (LITU) King's College Hospital London UK; ^2^ University of Nottingham Nottingham UK; ^3^ Department of Dermatology King's College Hospital London UK; ^4^ Department of Histopathology King's College Hospital London UK; ^5^ Liver Histopathology Laboratory King's College Hospital London UK

**Keywords:** acute liver failure, drug reaction with eosinophilia and systemic symptoms, drug‐induced liver injury, human herpes virus‐6, jaundice, liver transplant

## Abstract

Drug reaction with eosinophilia and systemic symptoms (DRESS) syndrome can have insidious symptoms which may lead to acute liver failure and death. Prompt recognition, stopping offending drug, and initiating corticosteroid are the mainstay of treatment. Early involvement of a specialist liver unit is vital.

## Graphical abstract

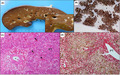

## 1 INTRODUCTION

We present a case of acute liver failure requiring emergency liver transplant due to trimethoprim‐induced drug reaction with eosinophilia and systemic symptoms (DRESS) and associated reactivation of human herpesvirus 6. The case highlights the importance of early recognition, stopping offending drug, and initiating corticosteroids to prevent the poor outcome.

Drug reaction with eosinophilia and systemic symptoms (DRESS) is a poorly recognized syndrome, which can lead to significant cutaneous, hematologic, and solid organ dysfunction. It may present with an insidious and nonspecific feature, leading to a delay in diagnosis. As this case illustrated, failure to recognize the condition in its earliest stages, stop the culprit drug, and initiate corticosteroid treatment may lead to a dramatically poor outcome for the patient. Our case outlines the clinical features of DRESS presenting to a physician, which can easily be mistaken for a viral illness. It highlights the role of acute liver failure units and emergency liver transplantation in severe cases progressing to liver failure. It also adds to the limited reports in the literature of positive outcomes following rescue liver transplantation in the context of severe DRESS.

## 2 CASE HISTORY AND EXAMINATION

A 22‐year‐old Caucasian woman had a 3‐week history of fluctuating fatigue, pyrexia, malaise, sweats, and myalgia presented to a local hospital with worsening symptoms, jaundice, and a macular rash starting on hands and feet that spread to rest of body. A week prior to hospitalization she also experienced nausea, vomiting, and diarrhea. She developed a diffused macular rash, marked liver function derangement, and significant coagulopathy. A presumptive diagnosis of acute liver failure secondary to viral infection or lymphoma was made, and the patient was transferred to the regional liver transplant unit for further management. On further evaluation by the receiving critical care team, it was established that the primary care physician had commenced her on trimethoprim for acne 6 weeks before the onset of symptoms. She had subsequently felt unwell within days of taking the medication and had discontinued it 2 weeks later. She had a history of Gilbert syndrome and well‐controlled asthma. She was a 3rd‐year medical student with no history of alcohol misuse, smoking, illicit drug use, foreign travel or other risk factors for liver disease. Her family history was insignificant. On physical examination, she was icteric, had a widespread macular rash affecting the trunk and limbs with marked facial and ear swelling. She had diffused axillary, cervical, and inguinal lymphadenopathy and hepatosplenomegaly. No genital, mucosal, or eye involvement was observed, though the patient was noted to have mild cheilitis. Her vital signs were stable; she was apyrexial with no overt signs of hepatic encephalopathy.

## 3 DIFFERENTIAL DIAGNOSIS, INVESTIGATIONS AND MANAGEMENT

**FIGURE 1:**
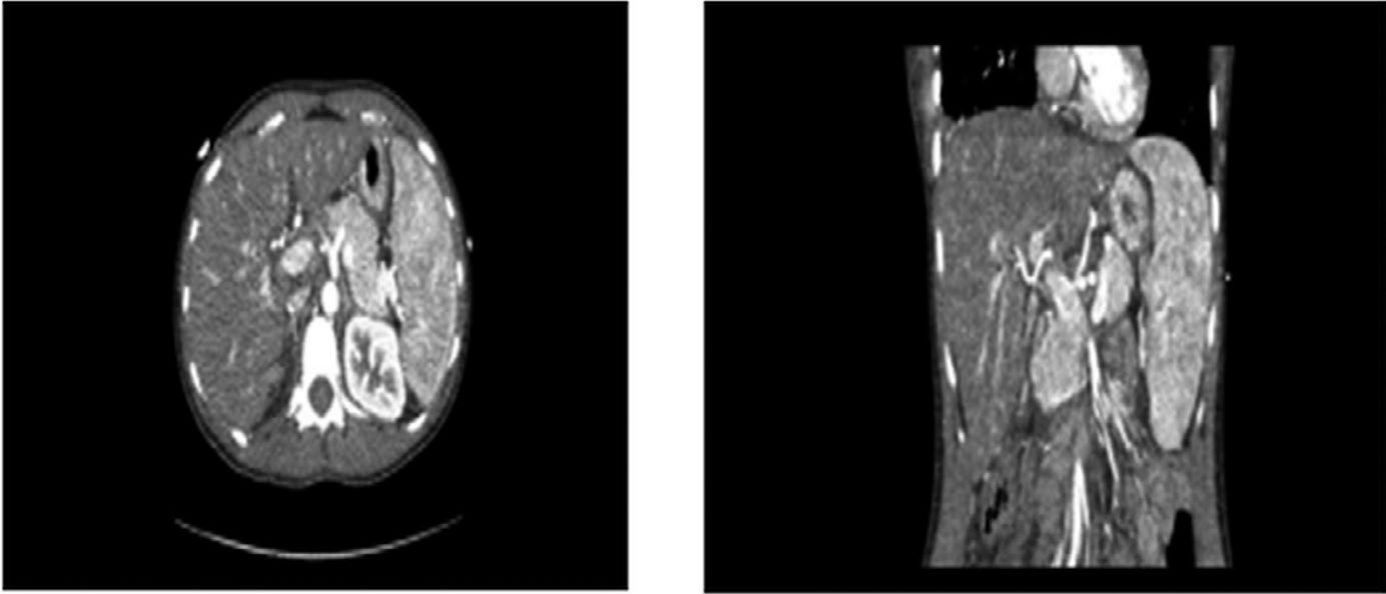
CT Abdomen: Lymphadenopathy with marked hepatosplenomegaly

**FIGURE 2:**
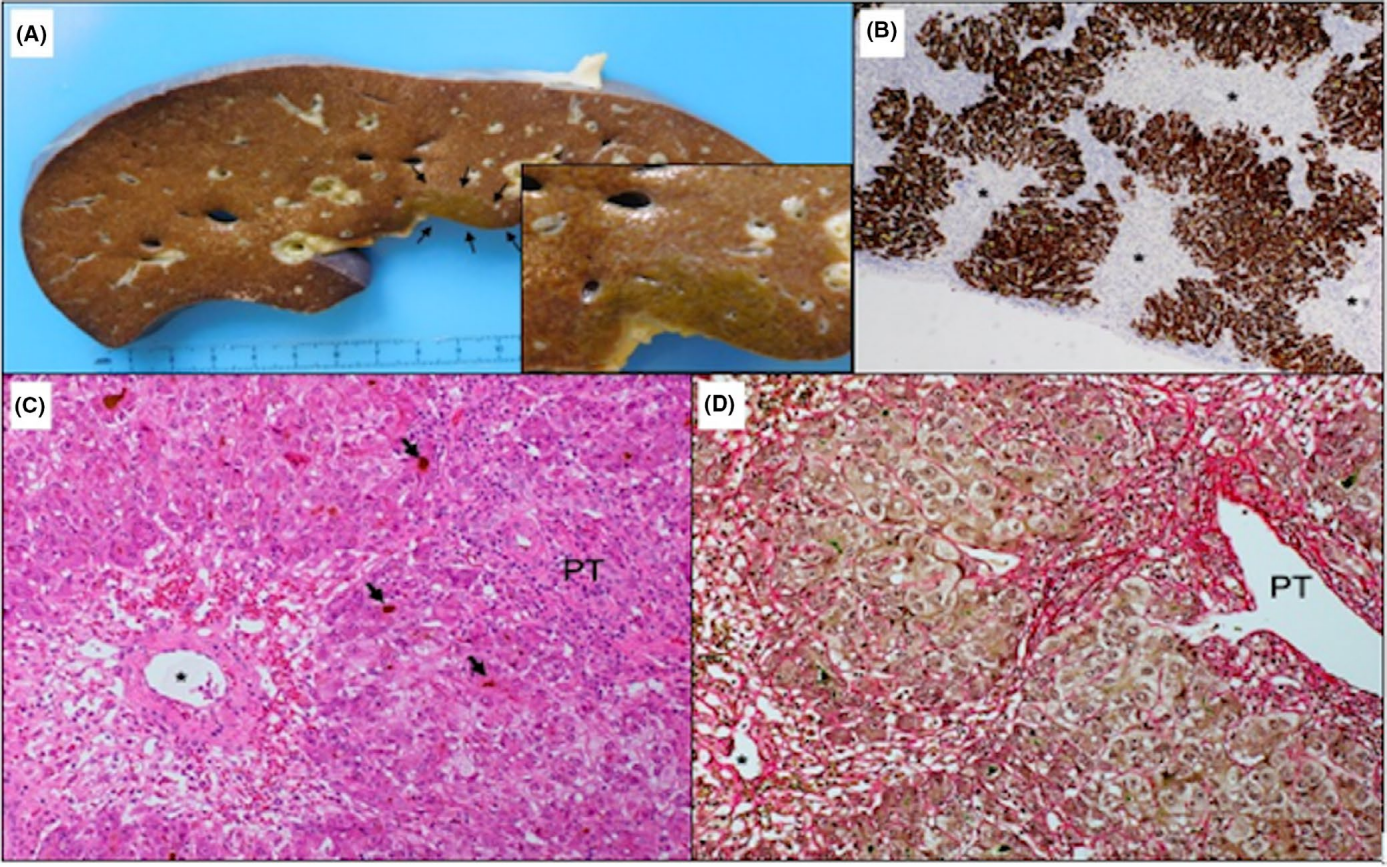
Liver histology: Subacute liver failure secondary to submassive hepatocellular necrosis. Representative macroscopic and microscopic images: A, Representative macroscopic section of the explanted liver. The liver is collapsed (723 g) with a single small area of residual parenchyma (inset), cholestatic (marked with arrows). B, Low magnification microscopy of this area demonstrates centrilobular‐based hepatocellular loss with bridging hepatocyte loss and collapse (*), in contrast with the total absence of hepatocytes observed in most of the liver tissue. Hepatocyte specific antigen immunostaining highlights the map‐like appearance of the area, 20×. C, Higher magnification of a centrilobular area, with perivenular confluent hepatocyte loss (*). Portal tracts (PT) show overall mild inflammation, with predominantly lymphocytes and occasional plasma cells and eosinophils. Residual hepatocytes show acute bilirubinostasis (arrows), H&E, 200×. D, Sirius red stain shows, no evidence of significant fibrosis, only collapse 200×

Day 1 of admission: In the context of the patient's history and presentation, the differential diagnoses of acute viral hepatitis, drug‐induced liver injury (DILI), drug rash with eosinophilia and systemic symptoms (DRESS), and lymphoproliferative disorder were considered. The results of admission investigations are given in Table 1, the full acute liver injury workup (Table 1) was initiated, and supportive management commenced as per the unit's set protocol. Day 2‐4 of admission: On the second day of admission to intensive care, she developed grade‐4‐hepatic encephalopathy along with hypotension, worsening renal dysfunction, hyperammonemia, and hyperlactatemia. Endotracheal intubation and mechanical ventilation for airway protection were required. She was deeply sedated for neurological protection, and vasopressor support was commenced. High volume continuous venovenous hemodiafiltration (CVVHDF) was initiated for ammonia clearance. Computer tomography (CT) of the liver (Figure 1), liver biopsy (Figure 2), skin, and lymph node biopsy was performed, the patient RegiSCAR score was calculated 7 (Table 1), and a diagnosis of DRESS syndrome was confirmed after early specialist input from dermatology, hepatology, and hematology. Under dermatology guidance, she was commenced on methylprednisolone 250 mg once a day, and although pulsed steroid therapy led to rapid resolution of the rash and pyrexia, and dramatic improvement transaminitis, her synthetic liver functions, jaundice, hepatic encephalopathy and physiology further deteriorated. She met poor prognostic criteria for spontaneous recovery without emergency liver transplantation. On the third day of admission, she was listed for super urgent liver transplantation with no contraindications. Although human herpes virus‐6 (HHV‐6) serology is not a routine screening test for transplant workup, a plasma sample had been requested due to its recognized association with DRESS. The results confirmed reactivation with a high viral load of >500 000 copies/mL. Antiviral treatment with intravenous acyclovir 10 mg/kg (IV) and intravenous foscarnet 15 mg/kg three times a day was subsequently commenced. Day 5 of admission: She received an orthotopic liver transplant. The explanted liver displayed histological features (Figure 2) most compatible with drug‐induced liver injury, hepatocellular type, consistent with the proposed diagnosis. However, as we could not demonstrate the presence or absence of the virus within the liver and there are no specific histological features for HHV6 infection, definitive exclusion of this virus as a direct cause of the hepatocellular damage is not possible. Immediate postoperatively she received a single 1 g dose of methylprednisolone followed by standard renal sparing immunosuppression with Prograf (tacrolimus) 2 mg twice a day together with a modified higher dose of methylprednisolone at 60 mg once a day as ongoing treatment for DRESS. Antiviral treatment was continued for HHV‐6 viremia. Day 1‐4 postoperative: After a transient rise in AST on postoperative day 2, her liver enzymes improved consistently. Day 5 postoperative: She was extubated. Day 7 postoperative: All organ support including renal replacement therapy was discontinued by, and she was discharged to the post‐transplant ward for further recovery and was eventually discharged home. Follow‐up: She continues to attend the post‐transplant follow‐up clinic and remains under dermatology follow‐up for management of acne with alternative agents and long‐term monitoring of DRESS.

**TABLE 1:** Laboratory investigation (non‐invasive acute liver failure screen)

Test	Result (Ref Range^a^)	Test	Result (Ref Range^a^)
Day 1 admission investigation			
Hemoglobin	127 (120‐160 g/L)	Total bilirubin	121 (3‐22 μmol/L)
Neutrophil	11.4 (2‐7.5 × 10^9^/L)	Conjugated bilirubin	84 (0‐5 μmol/L)
Total WCC	14.6 (4‐11 × 10^9^/L)	ALT	4143 (1‐50 IU/L)
Eosinophil	0.13 (0.1‐0.4 × 10^9^/L)	AST	5895 (1‐45 IU/L)
Platelets	106 (150‐400 × 10^9^/L)	ALP	246 (30‐130 IU/L)
INR	4.45 (0.9‐1.1)	GGT	140 (1‐54 IU/L)
Ammonia (NH4)	126 (11.2‐34.5 μmol/L)	Arterial PH	7.40 (7.35‐7.45)
LDH	2718 (<250 U/L)	Random glucose	5.2 (<11.1 mmol/L)
Serum Na	134 (135‐145 mmol/L)	HCO3	19.2 (22‐29 mmol/L)
Urea	12.5 (2.9‐8.2 mmol/L)	Lactate	8.2 (<2 mmol/L)
Serum creatinine	129 (50‐110 μmol/L)		
Peripheral blood film	Atypical lymphocytes^b^, toxic granulation and left shift of neutrophils		
Day 2 investigations			
Skin biopsy	Lymph node and bone marrow biopsy		Liver Biopsy (Figure 2)
CT AP (Figure 1)	Echocardiogram = normal		
Investigations sent on day 1, results were available on days 2 and 3			
Virology/microbiology panel			
Hepatitis A	IgG positive	Monospot	Negative
Hepatitis E	Negative	HHV IgM & IgG	Not done
Hepatitis BsAg & DNA	Negative	HHV‐6 DNA	555 879 copies/mL
Hepatitis C Ab	Negative	HHV‐8 DNA	Negative
CMV	IgG positive	VZV	IgG positive
EBV	IgG positive	Leptospira	Negative
Adenovirus	Negative	Toxoplasma	Negative
HIV	Negative	Atypical pneumonia	Negative
Other non‐invasive liver screens			
ANA	Negative	IgG	8.9 (6‐16 g/L)
ANCA	1/320 speckled	IgM	2.85 (0.4‐2.5 g/L)
DsDNA	Negative	IgA	1.77 (0.8‐3 g/L)
Anti‐GP Ab	Negative	HFE (H63D/C282Y)	Negative
Anti‐SM Ab	Negative	A1AT phenotype	PIMM (normal)
Anti‐LKM Ab	Negative	Ceruloplasmin	0.18 (0.2‐0.45 mg/dL)
Anti‐mitochondrial Ab	Negative	Ferritin	28 898 (41‐400 μg/L)
TSH	0.19 (0.27‐4.2 mIU/L)	Blood group	O**^+ (positive)^**
RegiSCAR score			
Patient RegiSCAR score = 7 Extent of rash = 1, rash suggestive of drug reaction = 1, systemic features = 4 (eosinophilia, lymph nodes, solid organ involvement, and fever), relevant negative tests (eg, hepatitis A, B, C) = 1 (RegiSCAR classification for likelihood diagnosis of DRESS: possible case score = 2 to 3; probable case score = 4 to 5; definite case score = 6 to 7).			

Abbreviations: A1 AT, alpha 1 antitrypsin; Ab, antibody; Ag, antigen; ALT, alanine transaminase; ANA, antinuclear antibody; ANCA, antinuclear cytoplasmic antibody; anti‐LKM, anti–liver‐kidney microsomal antibody; anti‐sm, antismooth muscle antibody; AST, aspartate transaminase; CMV, cytomegalovirus; Ds DNA, double‐stranded DNA; EBV, Epstein‐Barr virus; GGT, gamma‐glutamyl transferase; HCO3, bicarbonate; HFE, gene for hereditary hemochromatosis; HHV, human herpes virus; Ig, immunoglobulin; INR, international normalized ratio; LDH, lactate dehydrogenase; Na, sodium; NH4, ammonia; TSH, thyroid‐stimulating hormone; WCC, white cell count.

^a^ UK laboratory reference ranges for adult female.

^b^ Typical for DRESS.

## 4 DISCUSSION

Drug reaction with eosinophilia and systemic symptoms (DRESS) represents a severe idiosyncratic cutaneous response to medications that also have systemic involvement in the form of hematologic and solid organ dysfunction.^1^ Most commonly involved solid organs include the lungs (5%‐25% of cases), the kidneys (10%‐30% of cases), and the liver (60%‐80% of cases).^2^ Patients are often asymptomatic with deranged liver enzymes but may present with hepatomegaly and jaundice.^3^ There is usually a latency period between 2 and 6 weeks from the time a medication is started to development of symptoms.^4^ This long latency and the insidious and nonspecific symptoms in the early stages of DRESS lead to delays in diagnosis, and misattribution of symptoms to infection. Clinical features of DRESS itself include skin reactions and an inflammatory systemic involvement. DRESS generally begins with the eruption of a maculopapular skin rash symmetrically distributed over the body and extremities along with systemic symptoms including fevers, lymphadenopathy, and malaise.^5^

A scoring system for DRESS has been described by the European Registry of Severe Cutaneous Adverse Reactions (RegiSCAR).^6^ The RegiSCAR DRESS scoring system allows assessment of the probability that a particular constellation of clinical symptoms represents DRESS, as opposed to an alternative diagnosis such as infection, from a possible total score of 7, scores equate to the following probabilities: score of 2—3: possible case; score 4—5: probable case; and score 6—7: definite case.^6^ This scoring system includes acute rash, suspected drug‐related reaction, hospitalization, fever, laboratory abnormalities (lymphocyte count above or below normal, low platelet count, and/or eosinophilia), at least one internal organ involvement, and lymphadenopathy at more than two sites.^5^

Drugs commonly associated with DRESS include anticonvulsants like carbamazepine, phenytoin, and lamotrigine as well as antibiotics such as dapsone, minocycline, and vancomycin.^7^ There have also been cases of co‐trimoxazole as the causative agent in the development of DRESS.^8^ There appears to be an association between human leukocyte antigen (HLA) haplotypes and susceptibility to different drug exposures resulting in DRESS.^9^ Sukasem et al recently published a study suggesting that for the combination drug, co‐trimoxazole (CTX), which contains trimethoprim, there is an HLA susceptibility type for severe drug reactions—HLA‐B*15:02 and HLA‐C*08:01 alleles were significantly associated with CTX‐induced Stevens‐Johnson syndrome (SJS) and toxic epidermal necrolysis (TEN), and HLA‐B*13:01 alleles were significantly associated with CTX‐induced DRESS. However, this association was observed only in HIV‐infected patients but not in non‐HIV‐infected patients. Our patient was HIV negative so while it would be interesting to know if she was positive for HLA‐B*13:01, no conclusions could be drawn from this.^10^

Liver injury can be cholestatic, hepatocellular, or mixed and is defined by the RegiSCAR as serum alanine aminotransferase (ALT) levels more than two times the upper limit of normal and/or serum alkaline phosphatase (ALP) levels >1.5 times the upper limit of normal.^6^ Hematologic abnormalities are often detected during laboratory investigations and include leukocytosis, lymphocytosis, and eosinophilia.^2^ Although liver involvement is usually mild and transient, rarely, severe impairment progressing to acute liver failure (ALF) with jaundice, coagulopathy, and hepatic encephalopathy (HE) can occur.^7^ Severe liver injury is a contributing factor for having a prolonged clinical course of DRESS.^8^ In fact, severe hepatitis and liver failure accounts for most of the deaths associated with DRESS.^9^, ^10^

Tohyama et al^11^ looked at 100 patients with DRESS and found that patients with elevated HHV6 IgG antibodies suffered from severe organ involvement and a prolonged course compared with showing no reactivation of HHV‐6 and all patients with a fatal outcome were in the HHV‐6 reactivation group. A study by Harma et al^12^ found the presence of HHV‐6 in 80% of explanted livers in patients undergoing liver transplantation for ALF of unknown etiology.

Human herpesvirus reactivation especially human herpesvirus‐6 (HHV‐6) is frequently encountered which may suggest prior HHV‐6 infection as a risk factor for the development of DRESS.^4^ The immunological response against herpes viruses such as HHV‐6 by activated CD8+ T lymphocytes is implicated in the cause of the visceral and cutaneous manifestations, with higher cytokines levels in patients with the most severe visceral involvement. Severe manifestations of DRESS responsible for multiorgan failure or death have been seen in cases associated with an identification of HHV 6 reactivation. In the absence of DRESS, HHV‐6 alone as primary infection or reactivation has also been implicated in the cause of unexplained ALF in immunocompetent adults. Most cases of DRESS have been reported in adults with an incidence of 1 in 1000 to 1 in 10 000 drug exposures. The mortality rate is estimated to be between 5% to −10%.^13^, ^14^ The development of autoimmune conditions, particularly thyroid disease, but also diabetes mellitus, and alopecia areata following an episode of DRESS are well‐described. Patients should have thyroid function assessed 4‐6 weeks postrecovery, and at 6 months, or when symptoms suggestive of thyroid dysfunction present.^15^, ^16^

In our case, the patient had insidious fluctuating symptoms for weeks, which delayed her presentation, diagnosis, and initiation of definitive treatment. She had classical signs for DRESS and a high HHV6 viral load (viral DNA = 555 879 copies), which is in keeping with previously reported literature, where DRESS is frequently associated with human herpesvirus reactivation, especially HHV‐6. This may play a role in the development of severe liver injury and liver failure.

## 5 CONCLUSION

Overall, DRESS is a rare idiosyncratic drug reaction. The liver is the most commonly affected internal organ. Management of DRESS no matter the severity or type of organ involvement typically requires withdrawal of the suspected offending medication, oral or intravenous corticosteroids, and supportive care. Although liver involvement is usually mild and transient, severe organ dysfunction and ALF can occur requiring intensive supportive management. Corticosteroids may not be beneficial once ALF ensues and urgent liver transplantation is the only lifesaving option. Given the rarity of such cases, it is currently difficult to predict the prognosis post–liver transplantation for these patients. The early transfer to the liver transplant center after index suspicion, prompt involvement of relative specialities, a multidisciplinary approach and timely completion of investigations added to a positive outcome in our case.

## 6 LEARNING POINTS

Drug reaction with eosinophilia and systemic symptoms (DRESS) represents a severe idiosyncratic cutaneous response to medications that also have systemic involvement in the form of hematologic and solid organ dysfunction.

DRESS is often associated with human herpes virus‐6 (HHV‐6) reactivation.

The liver is the most common internal organ involved in DRESS. This is usually mild and transient but rarely can progress to severe impairment and acute liver failure (ALF).

Systemic high‐dose corticosteroids are the main treatment option.

If liver involvement progresses to severe hepatic dysfunction and ALF, corticosteroids may be of limited benefit and ultimately, lifesaving urgent liver transplantation is required.

## CONFLICT OF INTEREST

None declared.

## AUTHOR CONTRIBUTIONS

MS: developed case report concept and design, consented, collected the data, performed literature search, performed critical analysis, drafted the manuscript, and revised and wrote the final manuscript. VD: performed literature search and revised the manuscript. TP: revised the manuscript, served as specialist input on hepatology and liver intensive care aspect of the case report, and supervised the study. JS and RM: revised the manuscript and served as critical specialist input on histopathology aspect of the case report. SW and AC: revised the manuscript and served as critical specialist input on dermatology aspect of the case report.
